# Orofacial dyskinesia post resection of pineal gland tumor

**DOI:** 10.1093/jscr/rjaa465

**Published:** 2020-12-26

**Authors:** Mohamad Yazbeck, Baraa Dabboucy, Youssef Comair

**Affiliations:** Department of Neurosurgery, Faculty of Medicine, Lebanese University, Beirut, Lebanon; Department of Neurosurgery, Faculty of Medicine, Lebanese University, Beirut, Lebanon; Department of Neurosurgery, Lebanese Hospital Geitawi, Beirut, Lebanon

## Abstract

We reported a case of a 33-year-old lady who was diagnosed with a Pineal tumor and underwent craniotomy and gross total surgical resection of the mass through a right occipital transtentorial approach. Immediately upon extubation, the patient started to have persistent chewing-like movements typical of orofacial dyskinesia that resulted later in buccal mucosal injury and swelling of the lips. The movements spontaneously resolved after 3 days. The patient was not taking any medications that were known to induce such movements. Literature review showed that one of the possible mechanisms could be that the suddenly reduced melatonin level in the acute postoperative period leads to dysregulation of dopamine secretion in the nigrostriatal and limbic system causing these abnormal movements. To the best of our knowledge, this is the first such reported complication of orofacial dyskinesia post craniotomy for resection of the pineal tumor in humans.

## INTRODUCTION

In this paper, we report an exceptionally rare case of orofacial dyskinesia developing post craniotomy for resection of the pineal tumor. To the best of our knowledge, this complication has not been previously described post pineal tumor resection in humans. Albeit extremely rare, the review of the literature showed a possible correlation of the suddenly reduced melatonin level and the occurrence of such movements based on experiments done on rats.

## CASE REPORT

**Figure 1:**
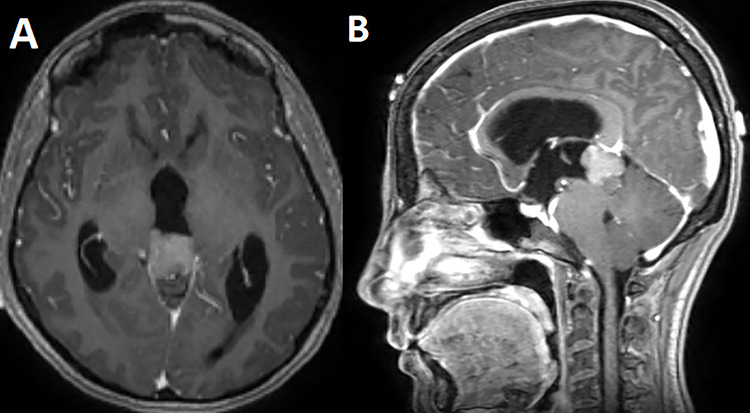
T1 enhanced axial (**A**) and sagittal (**B**) MRI showing a T1 hypointense homogenously enhancing pineal tumor.

**Figure 2:**
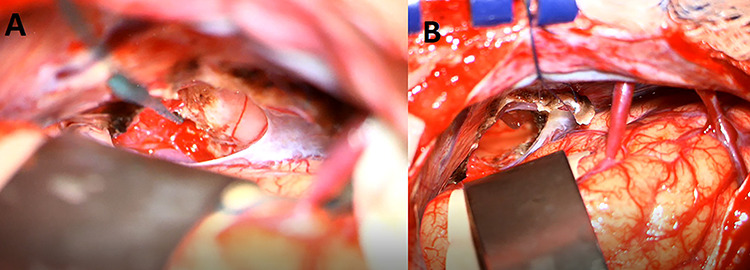
An intraoperative image under microscopic magnification showing the pineal tumor during the initial exposure (**A**) and the surgical cavity at the end of the gross total resection (**B**).

**Figure 3:**
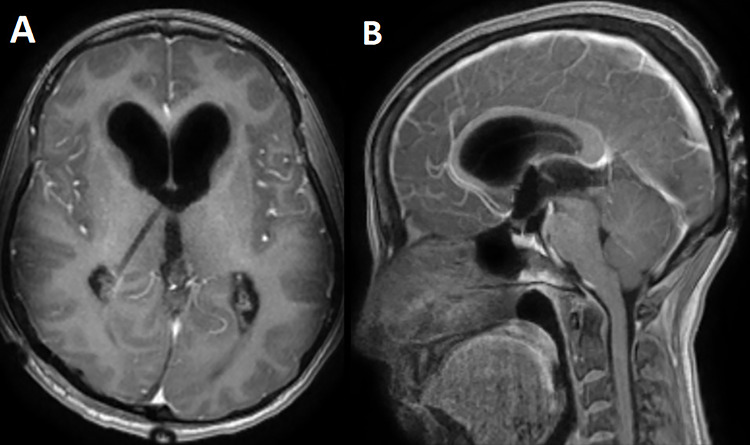
A postoperative day 1 T1 enhanced axial (**A**) and sagittal (**B**) MRI confirming complete surgical resection of the pineal tumor.

A 33-year-old lady presented to the clinic for bilateral retro-orbital headache with tinnitus most severe upon waking up in the morning for 3 months, without evidence of nausea, vomiting, or insomnia. Vital signs were within the normal range. The neurologic exam was non-focal. Enhanced brain magnetic resonance imaging (MRI) revealed a T1 hypointense, T2 hyperintense, homogenously enhancing Pineal tumor of 2 × 1.89 × 1.61 cm (anteroposterior × transverse × height) compressing the posterior wall of the third ventricle with evidence of moderate hydrocephalus (Fig. 1). The patient was scheduled for craniotomy for resection of the mass. In the operating room, an external ventricular drain was inserted in the right occipital horn followed by craniotomy and resection of the mass through a right occipital transtentorial approach. Gross total surgical resection was achieved under microscopic navigation (Fig. 2). The patient was extubated. Upon extubation the patient started to have chewing-like movement of her lips on the ET tube, afterward, she was transferred to the intensive care unit for monitoring. On postoperative day 1, we noticed that the patient was still having chewing-like movements typical of orofacial dyskinesia with evidence of buccal mucosal injury and swelling of the lips (Video 1). She was also complaining of inability to sleep. The patient was on regular postoperative medications (esomeprazole, cefazolin, paracetamol, morphine, dexamethasone, levetiracetam). An enhanced postoperative brain MRI was done for evaluation and confirmed complete resection of the lesion without any bleeding or ischemic changes (Fig. 3). The chewing like movements lasted for 72 hours and spontaneously resolved afterward. Pathology confirmed pineal parenchymal tumor of intermediate differentiation (WHO Grade III). The patient was treated with adjuvant radiotherapy. This is an unreported complication post pineal gland tumor resection in humans. Below we review the literature reporting abnormal facial movements associated with various pineal gland conditions as the data remains scarce concerning dyskinesia post pineal tumors resection in humans.

## DISCUSSION

Many drugs were associated with involuntary movement disorders mainly tardive dyskinesia; these drugs include antipsychotics (e.g. haloperidol), anticholinergics, antidepressants (e.g. fluoxetine, trazodone, clomipramine, lithium, ... ), monoamine oxidase inhibitors, antiemetics (e.g. metoclopramide), anticonvulsant (e.g. carbamazepine, phenytoin, …) and others like antihistamines, antiparkinsonian drugs, stimulants, decongestants and antimalarials [1].

Among several hypotheses about the pathophysiology drug-induced movement disorders, the blockade of dopamine receptors by dopamine antagonists is the most widely accepted theory [2].

In drug-naive rats, pinealectomy was reported to produce an increased incidence and severity of spontaneous perioral chewing movements compared to normal controls. These movements were augmented by the administration of haloperidol, suggesting that reduced melatonin secretion may be associated with the emergence of neuroleptic-induced dyskinesias and that the extrapyramidal side effects of neuroleptics may be in part mediated through the Pineal Gland [3].

Since pinealectomy produced increased spontaneous chewing movements in rats, reduced melatonin secretion may be associated with the pathophysiology of human Tardive dyskinesia and drug-induced dystonic movements. There is evidence to suggest a relationship between the presence of pineal calcification (PC) and melatonin secretion [4, 5]. Reduced melatonin secretion is expected to be associated with a diminished cerebral 5-HT activity which may ultimately contribute to the emergence of drug-induced dystonias [6]. There is evidence to suggest that melatonin regulates striatal and limbic dopamine (DA) activity where it has been reported to inhibit DA release in the hypothalamus, limbic system and retina [7, 8]. Thus, the significant association between enlarged PC and the presence of axial dyskinesias suggests that the pathophysiology of the latter may be associated with reduced melatonin secretion [9, 10].

To the best of our knowledge, this is the first reported complication of orofacial dyskinesia post craniotomy for resection of pineal tumor in humans. Our patient took regular perioperative medications that are not reported to cause any movement disorder. The proposed mechanism after reviewing the literature is that the suddenly reduced melatonin level in the acute postoperative period leads to dysregulation of dopamine secretion in the nigrostriatal and limbic system.

## CONFLICT OF INTEREST STATEMENT

None declared.

## FUNDING

None.

## INFORMED CONSENT

Written and signed informed consent was obtained from the patient.

## ETHICAL APPROVAL

Our institution does not require ethical approval for case reports.

## Supplementary Material

Video-1_rjaa465
